# Supplementary material for: Heuristic and optimal policy computations in the human brain during sequential decision-making
Source: Nat Commun. 2018 Jan 23;9:325. doi: 10.1038/s41467-017-02750-3 (PMC5780427; doi:10.1038/s41467-017-02750-3)
Supplement: Supplementary file 1 — Supplementary Information [file 41467_2017_2750_MOESM1_ESM.pdf]

Supplementary Information: Heuristic and optimal policy computations in the human brain during sequential decision-making

Supplementary Figures

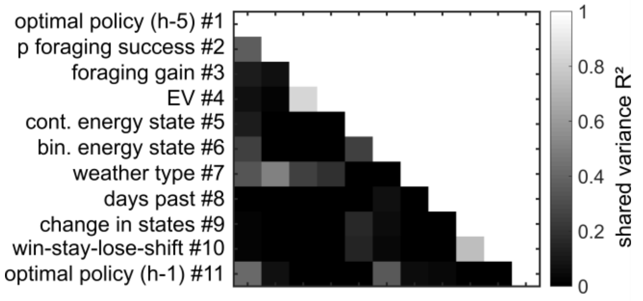

Supplementary Figure 1. Shared variances between optimal policy and heuristic variables
Shared variances were sufficiently low so that the 11 candidate variables were distinguishable in the employed task (mean shared variances derived from the fMRI sample). See Table 1 and Table 2 for lists of the variables and Supplementary Fig. 2 for plots illustrating how the optimal policy (at a horizon five time steps) relates to the 10 considered heuristic variables. h-5: horizon of 5 days; h-1: horizon of 1 day; cont.: continuous; bin.: binary

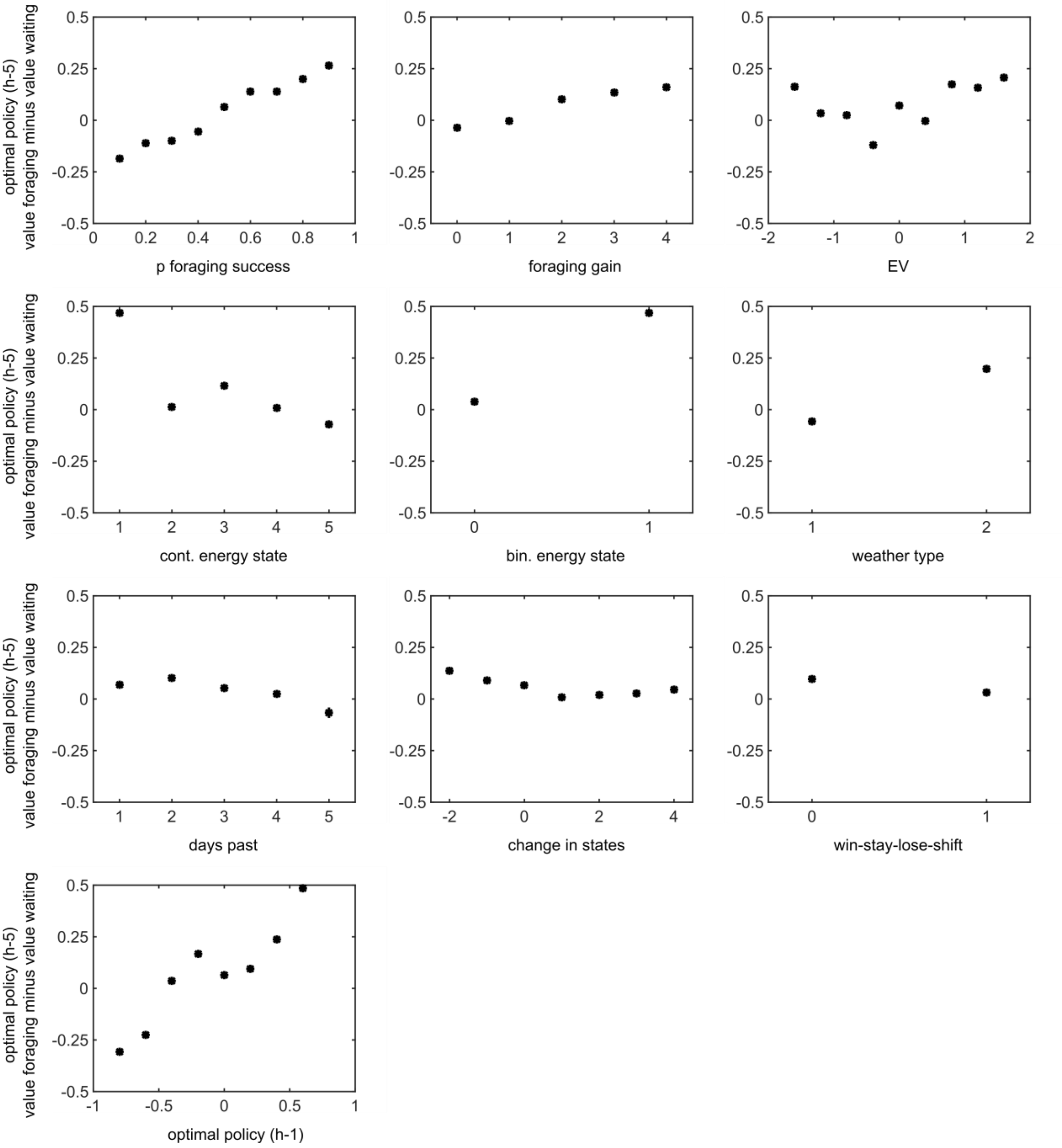

Supplementary Figure 2. Relation of optimal policy to heuristic variables
Relationship between the optimal policy (according to the normative horizon of five time steps) and all 10 heuristic variables considered. The probability of foraging success scales with the optimal policy. Data are binned and arranged in a similar same way as below in Supplementary Fig. 5 and Supplementary Fig. 9, which show the empirical relationship between data and fitted model. "Binary energy state," "weather type" and "wins-stay-lose-shift" are binary variables. See Table 1 and Table 2 for lists of the variables. h-5: horizon of 5 days; h-1: horizon of 1 day; cont.: continuous; bin.: binary

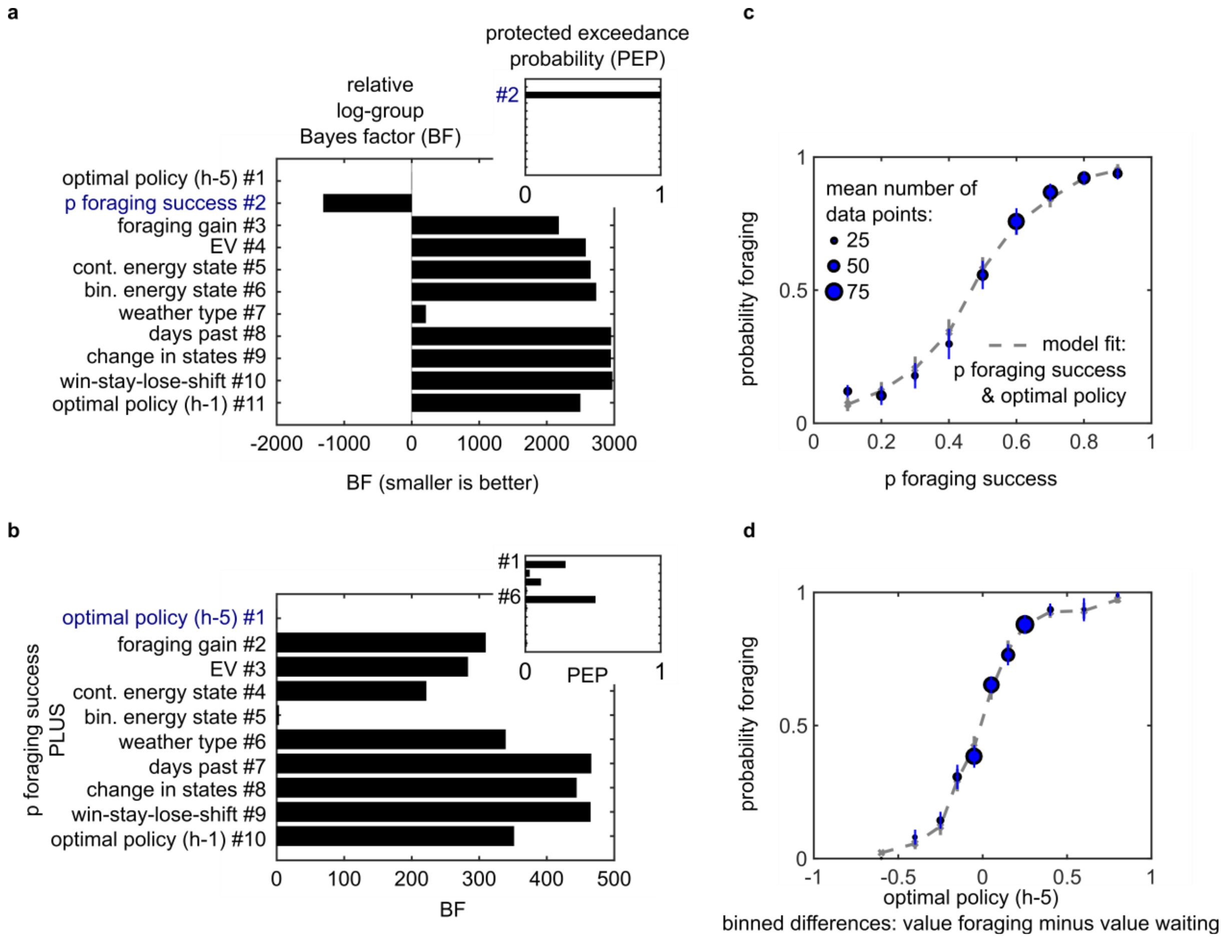

Supplementary Figure 3. Choice data and models of the behavioral sample
These plots show the choice data and models of the behavioral sample. The plots follow the same logic as those in Fig. 3, which show data and models of the fMRI sample.
(a) Models with a single predictor.
(b) Models with two predictors.
(c) Empirical data and winning model according to the probability of foraging success.
(d) Empirical data and winning model according to the optimal policy.
In the left-hand panels, blue font denotes the winning models. In the right-hand panels, error bars are SEM. Per data bin, circles depict mean empirical data points and lines and crosses depict mean model predictions (averaged for simulated data according to each participant's model fit). In several cases, error bars are smaller than the circles, which scale with the average number of trials contributing to the respective data points. See Supplementary Figure 4; Supplementary Figure 6; Supplementary Figure 7; Supplementary Figure 8; and Supplementary Figure 9 for further posterior predictive checks of the winning models and for parameter estimates of a full model including all candidate variables. See Supplementary Table 2 and Supplementary Table 4 for model comparisons in the behavioral sample.
h-5: horizon of 5 days; h-1: horizon of 1 day; cont.: continuous; bin.: binary

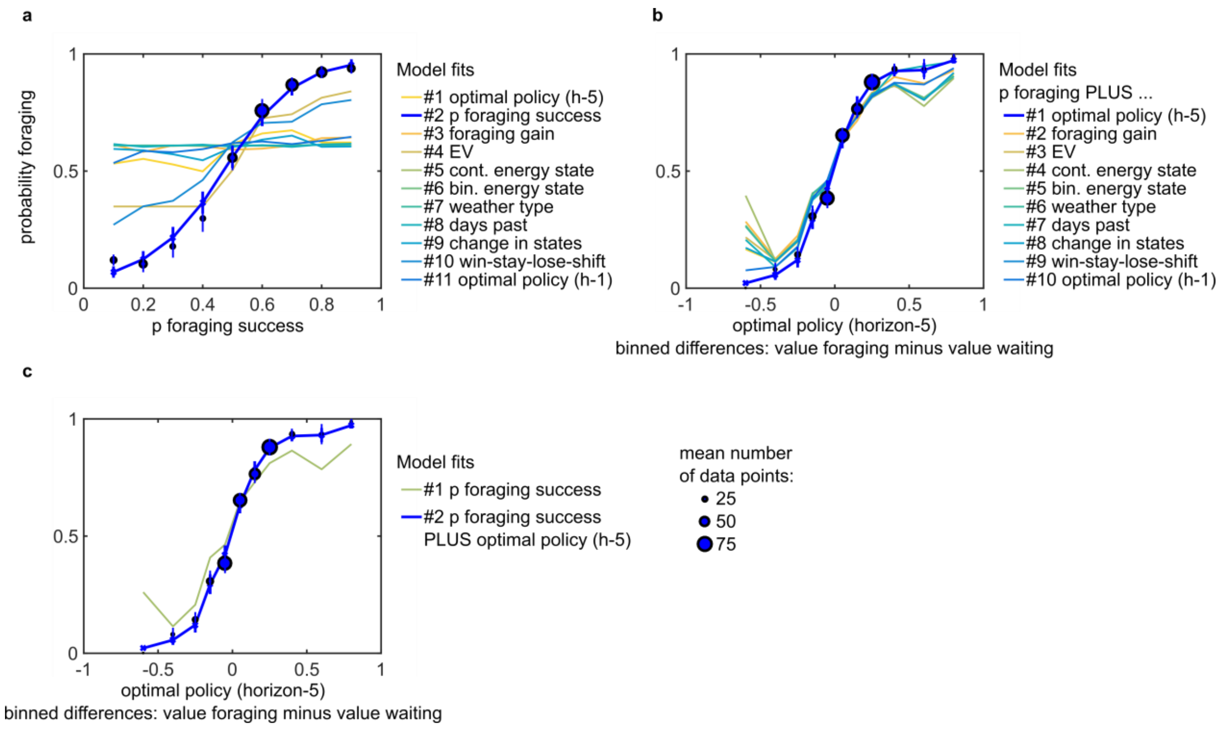

Supplementary Figure 4. Comparison of choice data to different model predictions in the behavioral sample

The plots follow the same logic as those in Fig. 4, which show data and models of the fMRI sample.

(a) Models with a single predictor.

(b) Models with two predictors.

(c) Model with a single predictor versus model with two predictors.

Error bars are SEM. Per data bin, circles depict mean empirical data points and colored lines depict mean model predictions (averaged for simulated data according to each participant's model fit). In several cases, error bars are smaller than the marker sizes, which scale with the average number of trials contributing to the respective data points. See Table 1 and Table 2 for lists of all variables. h-5: horizon of 5 days; h-1: horizon of 1 day; cont.: continuous; bin.: binary

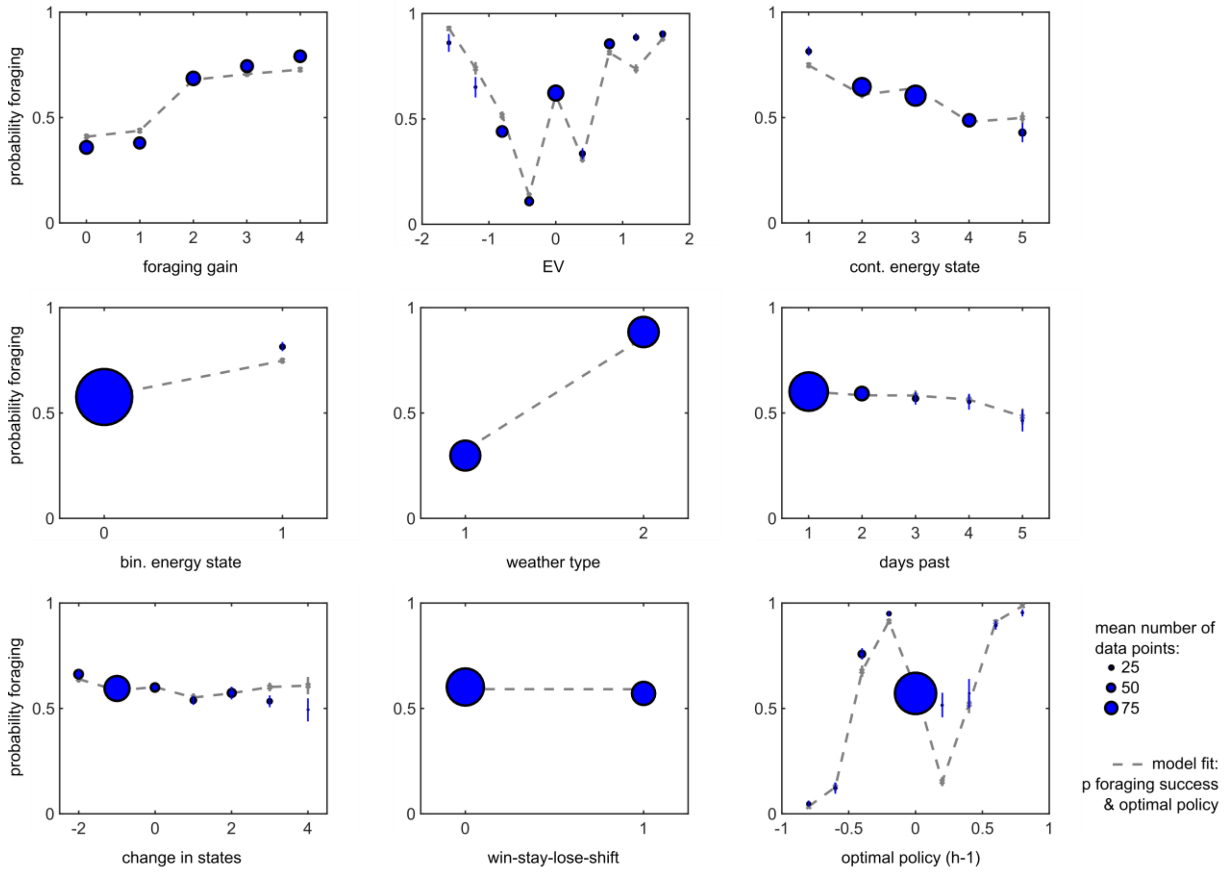

Supplementary Figure 5. Comparison of model predictions to choice data when splitting according to all candidate variables that did not win in the model comparisons (fMRI sample) Posterior predictive checks show that the winning model, which includes the probability of foraging success and the optimal policy, captures the empirical relationship between choice data and all other nine heuristic variables. “Binary energy state,” “weather type,” and “wins-stay-lose-shift” are binary variables. Error bars are SEM. Per data bin, circles depict mean empirical data points and dashed lines depict mean model predictions (averaged for simulated data according to each participant’s model fit). In several cases, error bars are smaller than the marker sizes, which scale with the average number of trials contributing to the respective data points. See Supplementary Fig. 9 for the same plots for the behavioral sample. See Table 1 and Table 2 for lists of all variables. h-5: horizon of 5 days; h-1: horizon of 1 day; cont.: continuous; bin.: binary

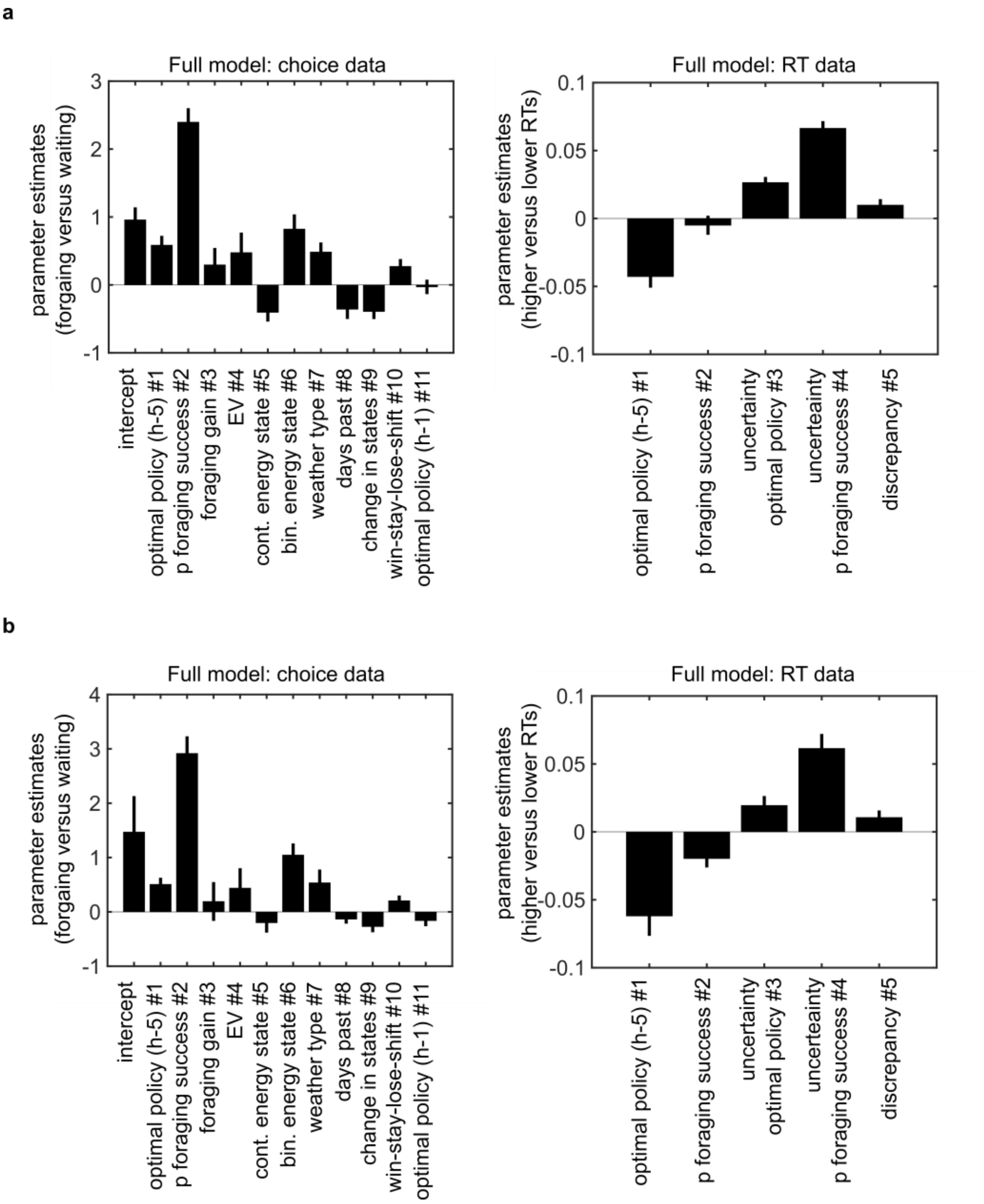

Supplementary Figure 6. Parameter estimates for full choice model including all candidate variables and parameter estimates for RT model (both samples)

(a) fMRI sample.

(b) Behavioral sample.

Mean parameter estimates of full behavioral and RT models that include all 11 or 5 candidate decision variables (which were z-scored). For better visualization the intercept for the RT model is not depicted. Mean parameter estimates were derived by averaging across parameter estimates of models fitted to individual participants. Error bars are SEM. h-5: horizon of 5 days; h-1: horizon of 1 day; cont.: continuous; bin.: binary

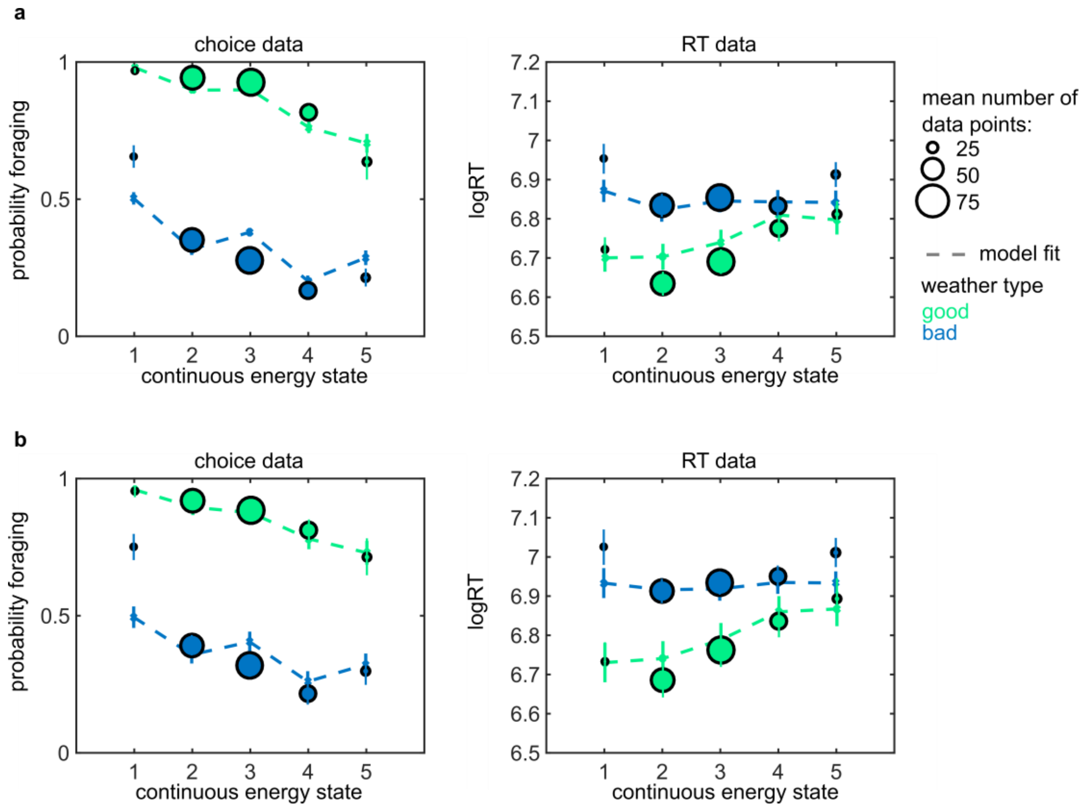

Supplementary Figure 7. Comparison of choice and RT data to model predictions when splitting conditions for continuous energy state and weather type (both samples)

(a) fMRI sample.

(b) Behavioral sample.

These plots separate data and model predictions according to two task variables: Continuous energy state (abscissa) and weather type (color-coded). The plots illustrate the quality of fit for the choice model, which includes the probability of foraging success and the optimal policy at a horizon of 5 time steps (left panels), and the quality of fit for the RT model, which includes the same two variables as the choice model along with the respective choice uncertainties and the discrepancies in their prescriptions of the two variables (right panels). The models capture the data well. The only notable exception is the data bin that includes energy state one and the bad weather condition, for which limited data points are available. See Supplementary Fig. 8 for more detailed illustrations and analyses regarding choice data with respect to energy states. Per data bin, mean empirical data points are depicted as circles (size according to number of data points) and mean model predictions are depicted as dashed lines. h-5: horizon of 5 days

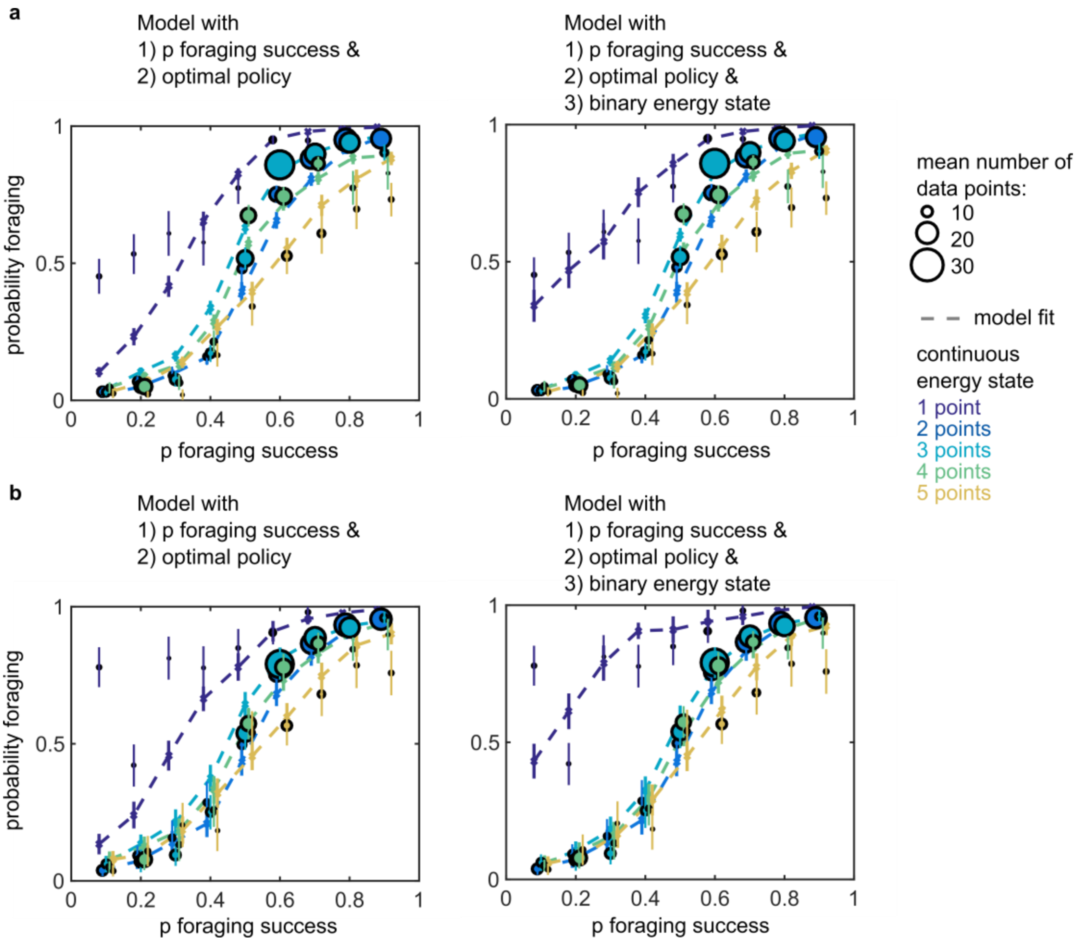

Supplementary Figure 8. Comparison of choice data to model predictions when splitting conditions for probability of foraging success and continuous energy (both samples)
(a) fMRI sample.
(b) Behavioral sample.
These plots separate data and model predictions according to two task variables: Probability of foraging success (abscissa) and continuous energy state (color-coded). The plots illustrate the quality of fit for the choice model, which includes the probability of foraging success and the optimal policy at a horizon of 5 time steps (left panels). This choice model captures the data well. Notable deviations of data and model fit are only observed for conditions with an energy state of one (and a rather low probability of foraging success). In our current study design, the data bin with energy state one only comprised a small fraction of trials (percentage: mean ± SD: fMRI sample: 0.07 ± 0.01; behavioral sample: 0.08 ± 0.02). For additional inspection, we provide plots for a choice model that includes the "binary energy state variable" (right panels). The more complicated model provides a better qualitative fit to the data bins with an energy state of one. However, the simpler model provides a decisively better model fit in the fMRI sample (Supplementary Table 1; Supplementary Table 3). In the behavioral sample, the model including binary energy state is decisively better according to log-group Bayes factors but not according to protected exceedance probabilities (Supplementary Table 2; Supplementary Table 4). Per data bin, mean empirical data points are depicted as circles (size according to number of data points) and mean model predictions are depicted as dashed lines.

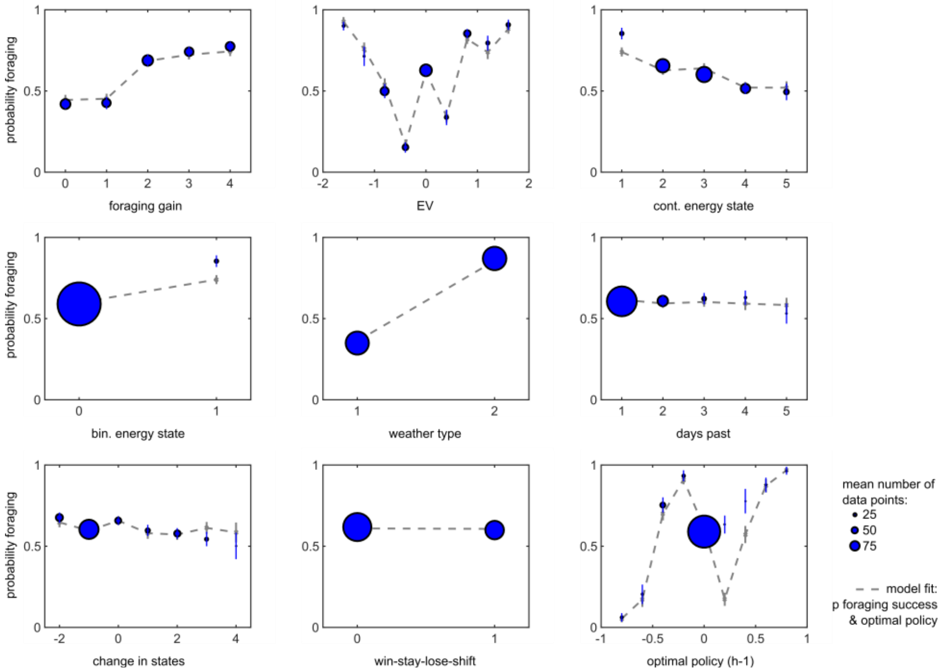

Supplementary Figure 9. Comparison of model predictions to choice data when splitting according to all candidate variables that did not win in the model comparisons (behavioral sample)

Same as Supplementary Fig. 5 but for the behavioral sample. Error bars are SEM. Per data bin, circles depict mean empirical data points and dashed lines depict mean model predictions (averaged for simulated data according to each participant's model fit). In several cases, error bars are smaller than the marker sizes, which scale with the average number of trials contributing to the respective data points. See Table 1 and Table 2 for lists of all variables. h-5: horizon of 5 days; h-1: horizon of 1 day; cont.: continuous; bin.: binary

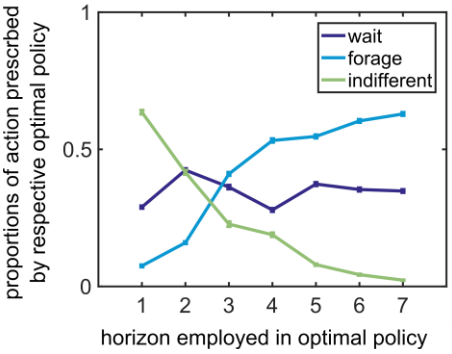

Supplementary Figure 10. Prescriptions of optimal policy according to different time horizons
This plot illustrates that the optimal policy makes differential prescriptions according to the time horizon employed. In the trials used in our task, the optimal policy at shorter time horizons is often indifferent between the two choice options of waiting versus foraging (e.g., when starvation is not possible on the next time step under any of the two options, a myopic optimal policy with a horizon of only 1 time step is indifferent).

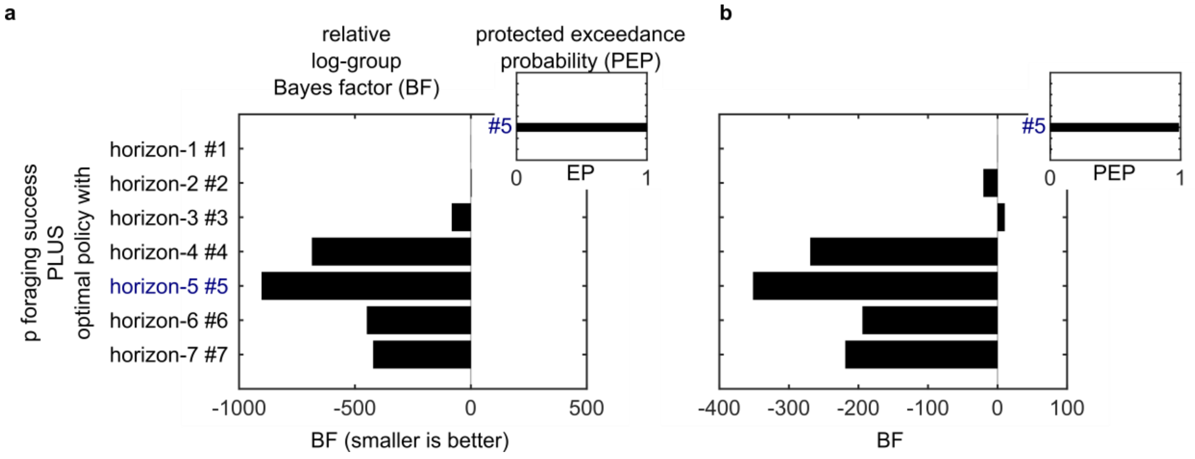

Supplementary Figure 11. Model comparisons for different time horizons of the optimal policy (both samples)
(a) fMRI sample.
(b) Behavioral sample.
Follow-up Bayesian model comparison suggested that participants used the time horizon of five days (highlighted in blue) that was normative in our task. Main plots depict fixed-effects analyses using log-group Bayes factors (based on Bayesian Information Criterion, BIC) relative to model #1. Insets show random-effects analyses using protected exceedance probabilities (PEP) with the winning model marked. See Supplementary Fig. 10 for an illustration of the different prescriptions according to the optimal policy with different time horizons.

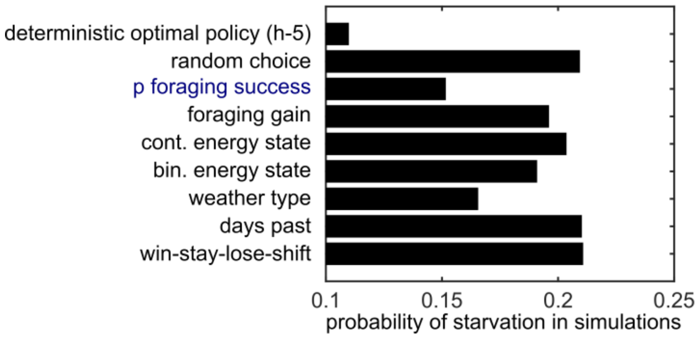

Supplementary Figure 12. Simulations of the probability of starvation according to the optimal policy and different heuristics

Simulations showed that the probability of foraging (highlighted in blue) constituted the best tested heuristic to minimize the probability of starvation. The two first bars show the benchmarks for the task: Strictly deciding according to the optimal policy resulted in the lowest probability of starvation whereas random choice resulted in the highest probability of starvation.

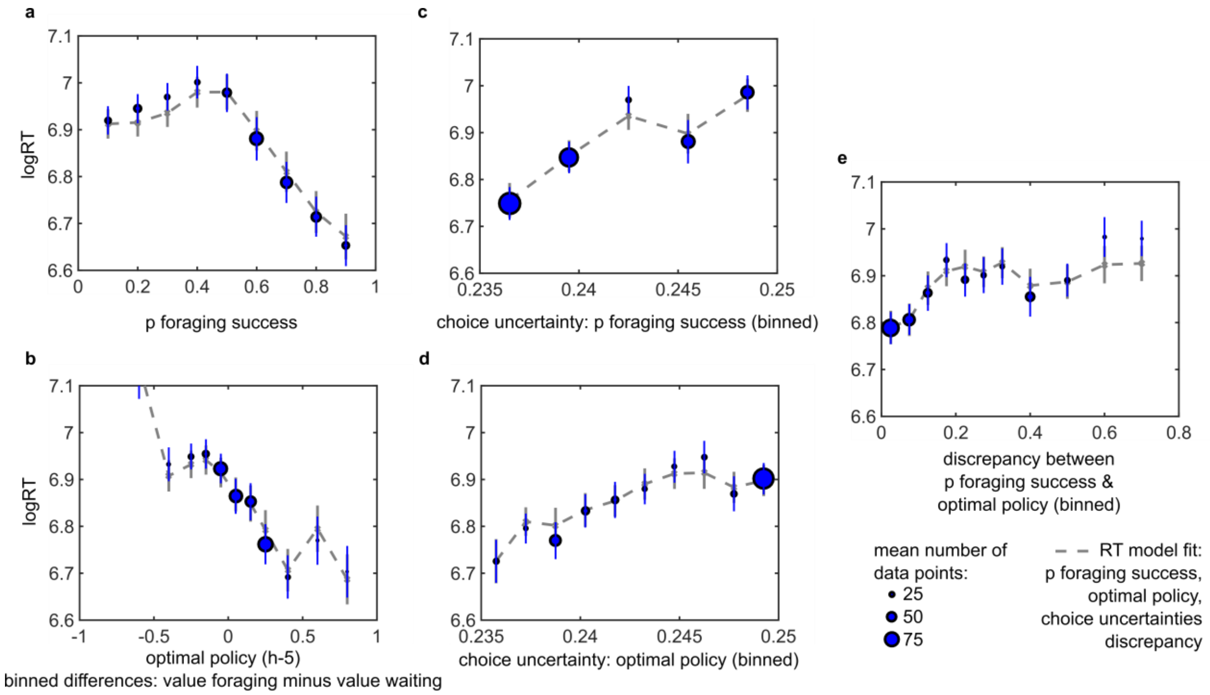

Supplementary Figure 13. Reaction time data and models of the behavioral sample
These plots show the RT data and models of the behavioral sample. The plots follow the same logic as those in Fig. 5, which show data and models of the fMRI sample.
(a) Probability of foraging success.
(b) Optimal policy.
(c) Choice uncertainty: Probability of foraging success.
(d) Choice uncertainty: Optimal policy.
(e) Discrepancies in prescriptions between probability of foraging success and optimal policy.
Error bars are SEM. Per data bin, circles depict mean empirical data points and lines and crosses depict mean model predictions (averaged for simulated data according to each participant's model fit). Circles scale with the average number of trials contributing to the respective data points.

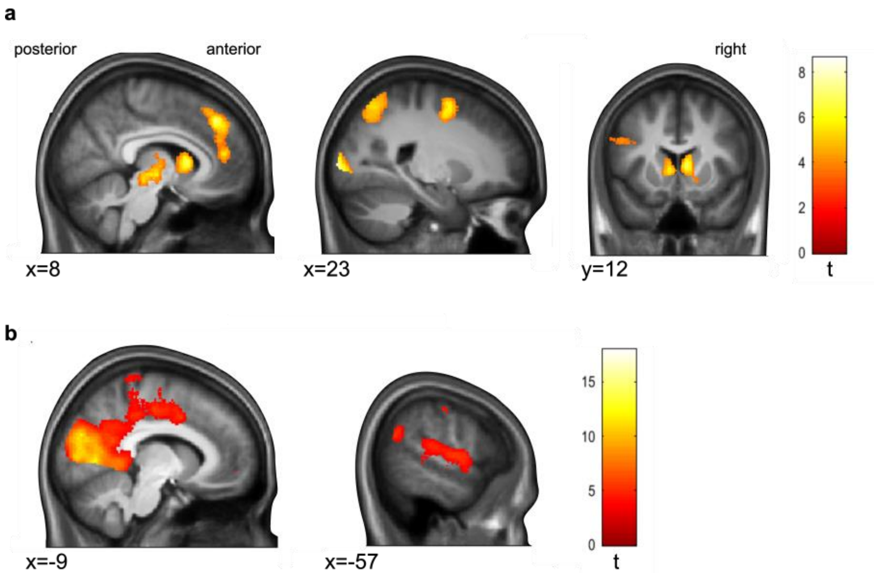

Supplementary Figure 14. Statistical parametric maps for the choice phase (GLM with participants' choices as only parametric modulator)

(a) Foraging > waiting: Amon other regions, DMPFC (x;y;z(MNI) = 8; 38; 42; t = 7.47), bilateral striatum (left: x;y;z(MNI) = -5; -24; -5; t = 6.78; right: x;y;z(MNI) = 8; 12; 6; t = 7.23), and DLPFC (x;y;z(MNI) = 23; 0; 56; t = 5.59) showed higher BOLD signals for foraging versus waiting.

(b) Waiting > foraging: Amon other regions, the medial occipital cortex extending into PCC (x;y;z(MNI) = -2; -81; 26; t = 17.996), the Rolandic operculum extending into superior temporal gyrus (x;y;z(MNI) = -45; -17; 0; t = 6.24), and the angular gyrus (x;y;z(MNI) = -57; -62; 24; t = 5.47) showed higher BOLD signals waiting versus foraging.

Overlay on group average T1-weighted image in MNI space; clusters are whole-brain FWE corrected for multiple comparisons at $p < 0.05$ with a cluster-defining threshold of $p < 0.001$. Color bars depict t-values. See Supplementary Table 9 for a list of all clusters.

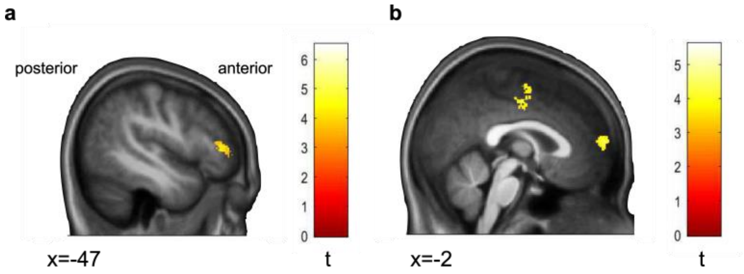

Supplementary Figure 15. Statistical parametric maps for the choice phase (GLM with values of chosen options as parametric modulator)
Depicted are results from a separate GLM, in which parametric modulators were framed in terms of the values of the chosen options (rather than in terms of the options presented).

(a) The probabilities of foraging success according to the chosen options (i.e., foraging or waiting) were positively related to the left frontal pole (x;y;z(MNI) = -47; 39; 6; t = 4.63; in a similar region as the probabilities of foraging framed in terms of the presented foraging option in the main GLM; i.e., x;y;z(MNI) = -42; 47; -5; t = 4.28).

(b) The values of the optimal policy according to the chosen options showed a positive relation with the VMPFC (x;y;z(MNI) = 2; 63; 11; t = 4.76; in a similar region as the differences between the presented choice options' values according to the optimal policy in the main GLM; i.e., x;y;z(MNI) = 6; 50; 6; t = 4.53).

Overlay on group average T1-weighted image in MNI space; clusters are whole-brain FWE corrected for multiple comparisons at $p < 0.05$ with a cluster-defining threshold of $p < 0.001$. Color bars depict t-values. See Supplementary Table 10 for a list of all clusters.

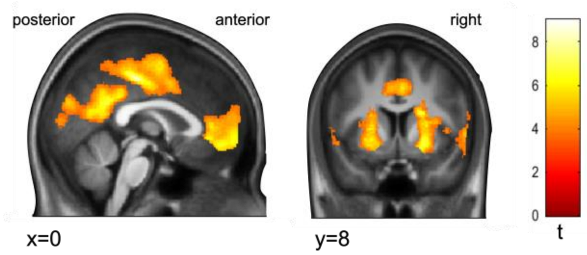

Supplementary Figure 16. Statistical parametric maps for the outcome phase (main GLM)
The overall peak voxel of the cluster is at the following location: x;y;z(MNI) = 0; -26; 48; t = 9.01. Overlay on group average T1-weighted image in MNI space; clusters are whole-brain FWE corrected for multiple comparisons at $p < 0.05$ with a cluster-defining threshold of $p < 0.001$. Color bars depict t-values. See Supplementary Table 11 for a list of all clusters.

Supplementary Tables

Supplementary Table 1. Comparison of choice models in fMRI sample

Model	Relative log-group Bayes factor (smaller is better)	Protected exceedance probability (larger is better)
**fMRI sample: Models with one variable (without interactions; see Fig. 3a)**		
#1 optimal policy (h-5)	0	0.016
#2 probabilities of foraging success	**-1230**	**0.984**
#3 foraging gain	3033	0
#4 EV	3607	0
#5 continuous energy state	3965	0
#6 binary energy state	4188	0
#7 weather type	204	0
#8 days past	4327	0
#9 change in states	4333	0
#10 win-stay-lose-shift	4378	0
#11 optimal policy (h-1)	3894	0
**fMRI sample: Models with two variables (without interactions; see Fig. 3b) – all models with probabilities of foraging success & …**		
#1 optimal policy (h-5)	**0**	**0.936**
#2 foraging gain	520	0.001
#3 EV	481	0.049
#4 continuous energy state	573	0
#5 binary energy state	501	0.012
#6 weather type	606	0
#7 days past	843	0
#8 change in states	828	0
#9 win-stay-lose-shift	875	0
#10 optimal policy (h-1)	902	0
**fMRI sample: Best model with one variable versus best model with two variables**		
#1 probability of foraging success	0	0
#2 probability of foraging success & optimal policy (h-5)	**-876**	**1**
**fMRI sample: Models with three variables (without interactions) – all models with probabilities of foraging success & optimal policy (h-5) & …**		
#1 foraging gain	0	0.052
#2 EV	**-60**	*0.438*
#3 continuous energy state	51	*0.231*
#4 binary energy state	94	0.125
#5 weather type	116	0.030
#6 days past	221	0.030
#7 change in states	206	0.030
#8 win-stay-lose-shift	231	0.028
#9 optimal policy (h-1)	180	0.036
**fMRI sample: Best model with two variables versus model with three variables**		
#1 probabilities of foraging success & optimal policy (h-5)	0	*0.451*
#2 probabilities of foraging success & optimal policy (h-5) & EV	**-216**	*0.549*
**fMRI sample: Best model with two variables versus models including interactions of probabilities of foraging success with other heuristic variables**		
#1 probabilities of foraging success & optimal policy (h-5)	**0**	**0.984**
#2 probabilities of foraging success, gain, & interaction	561	0.013
#3 probabilities of foraging success, continuous energy state,	546	0.001

& interaction		
#4 probabilities of foraging success, binary energy state, & interaction	581	0.002
#5 probabilities of foraging success, weather type, & interaction	629	0
#6 probabilities of foraging success, days past, & interaction	795	0
**fMRI sample: Best model with two variables versus models including interactions with choice uncertainties and discrepancy**		
#1 probabilities of foraging success & optimal policy (h-5)	0	0.521
#2 probabilities of foraging success * (1 - choice uncertainty of probabilities of foraging success) & optimal policy (h-5)	1	0.156
#3 probabilities of foraging success & optimal policy (h-5) * (1 - choice uncertainty of optimal policy)	1	0.125
#4 probabilities of foraging success * (1 - choice uncertainty of probabilities of foraging success) & optimal policy (h-5) * (1 - choice uncertainty of optimal policy)	2	0.080
#5 probabilities of foraging success * discrepancy & optimal policy (h-5)	1190	0.058
#6 probabilities of foraging success & optimal policy (h-5) * discrepancy	284	0.060

Among 11 candidate variables, probability of foraging success emerged as the single best predictor of participants’ choices. The model that included both the probabilities of foraging success and the optimal policy outperformed 1) all other combinations of probability of foraging success and the remaining 10 candidate variables, 2) the model with the probabilities of foraging success as sole predictor, and 3) models including the probabilities of foraging success, along with five different heuristics, and the respective interactions. No decisive evidence emerged for 1) models with a third candidate variable consistently explaining more variance (according to protected exceedance probabilities), and 2) a clear-cut influence of choice uncertainties or discrepancy between the two policies included in the main model (according to relative log-group Bayes factors and protected exceedance probabilities).

Statistics of winning models are printed in bold font. If protected exceedance probabilities do not decisively indicate a best model, the statistics of the two best models according to protected exceedance probability are printed in italics. See Fig. 3 for main model comparisons as well as Supplementary Fig. 3 and Supplementary Table 2 for the behavioral sample.

Supplementary Table 2. Comparison of choice models in behavioral sample

Model	Relative log-group Bayes factor (smaller is better)	Protected exceedance probability (larger is better)
**Behavioral sample: Models with one variable (without interactions; see Supplementary Fig. 3a)**		
#1 optimal policy (h-5)	0	0.001
#2 probabilities of foraging success	**-1308**	**0.999**
#3 foraging gain	2175	0
#4 EV	2574	0
#5 continuous energy state	2646	0
#6 binary energy state	2728	0
#7 weather type	206	0
#8 days past	2949	0
#9 change in states	2946	0
#10 win-stay-lose-shift	2968	0
#11 optimal policy (h-1)	2494	0
**Behavioral sample: Models with two variables (without interactions; see Supplementary Fig. 3b) – all models with probabilities of foraging success & …**		
#1 optimal policy (h-5)	**0**	*0.298*
#2 foraging gain	309	0.031
#3 EV	283	0.115
#4 continuous energy state	221	0.009
#5 binary energy state	3	*0.516*
#6 weather type	339	0.009
#7 days past	466	0.004
#8 change in states	444	0.004
#9 win-stay-lose-shift	465	0.004
#10 optimal policy (h-1)	351	0.009
**Behavioral sample: Best model with one variable versus best model with two variables**		
#1 probability of foraging success	0	0.089
#2 probability of foraging success & optimal policy (h-5)	**-436**	**0.911**
**Behavioral sample: Models with three variables (without interactions) – all models with probabilities of foraging success & optimal policy (h-5) & …**		
#1 foraging gain	0	0.023
#2 EV	-30	0.104
#3 continuous energy state	-44	*0.252*
#4 binary energy state	**-104**	*0.605*
#5 weather type	48	0.004
#6 days past	99	0.002
#7 change in states	112	0.002
#8 win-stay-lose-shift	118	0.002
#9 optimal policy (h-1)	1	0.006
**Behavioral sample: Best model with two variables versus model with three variables**		
#1 probabilities of foraging success & optimal policy (h-5)	0	*0.179*
#2 probabilities of foraging success & optimal policy (h-5) & binary energy state	**-149**	*0.821*
**Behavioral sample: Best model with two variables versus models including interactions of probabilities of foraging success with other heuristic variables**		
#1 probabilities of foraging success & optimal policy (h-5)	**0**	*0.349*
#2 probabilities of foraging success, gain, & interaction	344	0.260
#3 probabilities of foraging success, continuous energy state,	182	0.013

& interaction		
#4 probabilities of foraging success, binary energy state, & interaction	44	*0.368*
#5 probabilities of foraging success, weather type, & interaction	389	0.005
#6 probabilities of foraging success, days past, & interaction	425	0.005
**Behavioral sample: Best model with two variables versus models including interactions with choice uncertainties and discrepancy**		
#1 probabilities of foraging success & optimal policy (h-5)	0	0.198
#2 probabilities of foraging success * (1 - choice uncertainty of probabilities of foraging success) & optimal policy (h-5)	-1	0.147
#3 probabilities of foraging success & optimal policy (h-5) * (1 - choice uncertainty of optimal policy)	0	0.178
#4 probabilities of foraging success * (1 - choice uncertainty of probabilities of foraging success) & optimal policy (h-5) * (1 - choice uncertainty of optimal policy)	-1	0.144
#5 probabilities of foraging success * discrepancy & optimal policy (h-5)	1011	0.130
#6 probabilities of foraging success & optimal policy (h-5) * discrepancy	133	0.202

Among 11 candidate variables, probability of foraging success emerged as the single best predictor of participants' choices. The model that included both the probabilities of foraging success and the optimal policy outperformed 1) all other combinations of probability of foraging success and the remaining 10 candidate variables (according to relative log-group Bayes factors; the model including the binary energy state variable performed second-best), 2) the model with the probabilities of foraging success as sole predictor, and 3) models including the probabilities of foraging success, along with five different heuristics, and the respective interactions (according to relative log-group Bayes factors; the model including the binary energy state and the respective interaction performed second-best). No decisive evidence emerged for 1) models with a third candidate variable consistently explaining more variance (according to protected exceedance probabilities), and 2) a clear-cut influence of choice uncertainties or discrepancy between the two policies included in the main model (according to relative log-group Bayes factors and protected exceedance probabilities).

Statistics of winning models are printed in bold font. If protected exceedance probabilities do not decisively indicate a best model, the statistics of the two best models according to protected exceedance probability are printed in italics. See Supplementary Fig. 3 for main model comparisons as well as Fig. 3 and Supplementary Table 1 for the fMRI sample.

Supplementary Table 3. Comparison of choice models with all possible combinations of two candidate variables (fMRI sample)

	#1	#2	#3	#4	#5	#6	#7	#8	#9	#10	#11
Log-group Bayes factors based on Bayesian Information Criterion (BIC) relative to the model including #1 (the optimal policy (h-5) and #2 p foraging success (smaller is better)											
#1		**0**	1837	1925	2064	2045	992	2150	2149	2133	1954
#2			520	481	573	501	606	843	828	875	902
#3				4989	4674	4992	2352	5172	5174	5225	4835
#4					5294	5589	2379	5745	5748	5803	5348
#5						6156	1626	6107	6168	6155	5263
#6							2099	6235	6367	6389	6067
#7								2257	2302	2369	2313
#8									6471	6519	5976
#9										6565	6038
#10											6080
Protected exceedance probability (larger is better)											
#1		*0.840*	0	0	0	0	0	0	0	0	0
#2			0	*0.130*	0	0.020	0	0	0	0	0
#3				0	0	0	0	0	0	0	0
#4					0	0	0	0	0	0	0
#5						0	0	0	0	0	0
#6							0	0	0	0	0
#7								0	0	0	0
#8									0	0	0
#9										0	0
#10											0

Model comparison of a total of 55 models with all binary combinations of the 11 candidate decision variables. Log-group Bayes factors and indicate that winning model comprises the probability of foraging success and the optimal policy with a horizon of 5 time steps (protected exceedance probabilities also favor the same model).

Statistics of the winning model according to BIC are printed in bold font. Statistics of the two best models according to protected exceedance probability are printed in italics. See Fig. 3 and Supplementary Table 1 for main model comparisons and Supplementary Table 4 for the behavioral sample.

Variables:
#1 optimal policy (horizon-5)
#2 p foraging success
#3 foraging gain
#4 EV
#5 continuous energy state
#6 binary energy state
#7 weather type
#8 days past
#9 change in states
#10 win-stay-lose-shift
#11 optimal policy (horizon-1)

Supplementary Table 4. Comparison of choice models with all possible combinations of two candidate variables (behavioral sample)

	#1	#2	#3	#4	#5	#6	#7	#8	#9	#10	#11
Log-group Bayes factors based on Bayesian Information Criterion (BIC) relative to the model including #1 (the optimal policy (h-5) and #2 p foraging success (smaller is better)											
#1		**0**	1655	1722	1730	1732	1055	1773	1829	1814	1598
#2			309	283	221	3[a]	339	466	444	465	351
#3				3771	3608	3743	2015	3982	3982	4009	3605
#4					4041	4159	2014	4384	4381	4406	3957
#5						4343	1513	4463	4492	4476	3720
#6							1742	4527	4560	4558	4288
#7								2005	1993	2016	1850
#8									4758	4783	4303
#9										4755	4295
#10											4316
Protected exceedance probability (larger is better)											
#1		*0.270*	0	0	0	0	0	0	0	0	0
#2			0.030	0.110	0.010	*0.530*	0.010	0	0	0	0
#3				0	0	0	0	0	0	0	0
#4					0	0	0	0	0	0	0
#5						0	0	0	0	0	0
#6							0	0	0	0	0
#7								0	0	0	0
#8									0	0	0
#9										0	0
#10											0

Model comparison of a total of 55 models with all binary combinations of the 11 candidate decision variables. Log-group Bayes factors and indicate that winning model comprises the probability of foraging success and the optimal policy with a horizon of 5 time steps (protected exceedance probabilities do not decisively distinguish between this model and the model comprising the probability of foraging success and the binary energy state variable).

Statistics of the winning model according to BIC are printed in bold font. Statistics of the two best models according to protected exceedance probability are printed in italics. See Supplementary Fig. 3 and Supplementary Table 2 for main model comparisons and Supplementary Table 3 for the fMRI sample.

Variables:
#1 optimal policy (horizon-5)
#2 p foraging success
#3 foraging gain
#4 EV
#5 continuous energy state
#6 binary energy state
#7 weather type
#8 days past
#9 change in states
#10 win-stay-lose-shift
#11 optimal policy (horizon-1)
[a] Exact value > 3

Supplementary Table 5. Analyses of choice data in subsets of trials with opposing prescriptions according to different time horizons of the optimal policy (both samples)

Condition	Mean (SD) proportion of participants' choices to forage in respective conditions	t-values (test against midpoint of choice proportion, i.e., 0.5)	p-values	Mean (SD) proportion of trials in respective condition versus total number of trials
**fMRI sample**				
Foraging prescribed by horizon-5 optimal policy & waiting prescribed by …				
horizon-1 policy	0.94 (0.06)	38.45	$< 10^{-24}$	0.14 (0.012)
horizon-2 policy	0.79 (0.07)	21.57	$< 10^{-17}$	0.24 (0.014)
horizon-3 policy	0.82 (0.11)	15.79	$< 10^{-14}$	0.11 (0.010)
horizon-4 policy	0.71 (0.21)	5.23	$< 10^{-4}$	0.03 (0.007)
Waiting prescribed by horizon-5 optimal policy & foraging prescribed by …				
horizon-3 policy	0.20 (0.22)	-7.26	$< 10^{-7}$	0.02 (0.007)
horizon-4 policy	0.26 (0.22)	-5.96	$< 10^{-5}$	0.02 (0.006)
**Behavioral sample**				
Foraging prescribed by horizon-5 optimal policy & waiting prescribed by …				
horizon-1 policy	0.92 (0.17)	11.58	$< 10^{-9}$	0.14 (0.019)
horizon-2 policy	0.77 (0.15)	8.13	$< 10^{-7}$	0.24 (0.015)
horizon-3 policy	0.79 (0.20)	6.52	$< 10^{-5}$	0.12 (0.012)
horizon-4 policy	0.75 (0.24)	4.83	$< 10^{-3}$	0.03 (0.010)
Waiting prescribed by horizon-5 optimal policy & foraging prescribed by …				
horizon-3 policy	0.19 (0.25)	-5.60	$< 10^{-5}$	0.02 (0.005)
horizon-4 policy	0.39 (0.25)	-1.98	= 0.062	0.02 (0.005)

Participants' choices follow the optimal policy with a horizon of 5 time steps, which is normative in our task. That is, in all subsets of trials, in which different shorter horizons of the optimal policy made opposing prescriptions, choices were in line with the prescriptions of the optimal policy with a horizon of 5 steps. See Supplementary Fig. 10 for an illustration of the differential prescriptions of the optimal policy according to different time horizons and Supplementary Fig. 11 for formal model comparisons of choice data.

Supplementary Table 6. Model of log-transformed reaction times (both samples)

Predictor	t-value	p-value
**fMRI sample**		
Intercept	11.13	
Heuristic: Probability of foraging success (with higher numbers indicating higher probability)	-8.46	$< 10^{-8}$
Optimal policy (value differences of foraging versus waiting)	-0.77	> 0.4
Choice uncertainty of heuristic: Uncertainty of probability of foraging success (with higher numbers indicating higher uncertainty)	15.34	$< 10^{-15}$
Choice uncertainty of optimal policy (with higher numbers indicating higher uncertainty)	2.68	< 0.05
Discrepancy between heuristic and optimal policies (positive trial-by-trial relation with higher numbers indicating larger discrepancies)	6.55	$< 10^{-5}$
**Behavioral sample**		
Intercept	6.39	
Heuristic: Probability of foraging success (with higher numbers indicating higher probability)	-4.27	$< 10^{-3}$
Optimal policy (value differences of foraging versus waiting)	-3.00	< 0.01
Choice uncertainty of heuristic: Uncertainty of probability of foraging success (with higher numbers indicating higher uncertainty)	5.80	$< 10^{-5}$
Choice uncertainty of optimal policy (with higher numbers indicating higher uncertainty)	1.86	= 0.062
Discrepancy between heuristic and optimal policies (positive trial-by-trial relation with higher numbers indicating larger discrepancies)	2.94	< 0.01

Log-transformed RTs were analyzed using linear mixed effects models as implemented in the R package lmer [4]. Importantly, RTs were slower when discrepancies between the heuristic and the optimal policies were higher (see Table 1 and Table 2 for lists of the used variables). The same analyses using untransformed RTs resulted in a qualitatively identical pattern. See Fig. 5 and Supplementary Fig. 13 for illustration of RT data and models.

Supplementary Table 7. fMRI results during choice phase (main GLM)

	Side	Peak voxel MNI coordinates (mm) X	Y	z	Cluster size (Voxel)	Peak t score
**Heuristic: Probability of foraging success** (positive trial-by-trial relation with higher numbers indicating higher probability)						
Occipital lobe (calcarine fissure)	R	27	-95	8	649	6.85
Dorsolateral cortex (DLPFC)	L	-21	-5	60	1122	6.59
Superior parietal gyrus	L	-23	-65	63	1698	6.44
Dorsal MPFC (DMPFC) extending into pre-supplementary motor area (pre-SMA)	L/R	8	29	50	817	5.80
Intraparietal sulcus (IPS)	R	47	-42	50	2772	5.37
Inferior frontal gyrus (IFG)	R	29	27	-5	649	5.36
DLPFC	R	23	-3	50	691	5.19
Occipital lobe (calcarine fissure)	L	-32	-93	8	221	4.87
IPS	L	-42	-45	51	313	4.42
Frontal pole (middle frontal gyrus)	L	-42	47	-5	261	4.28
**Heuristic: Probability of foraging success** (negative trial-by-trial relation)						
Occipital lobe (calcarine fissure)	L/R	-2	-84	30	8113	8.42
Perigenual anterior cingulate cortex (ACC)/ ventral MPFC (VMPFC)	L/R	6	33	6	1056	6.27
Pallidum	L	-23	-2	-6	203	5.25
**Optimal policy** (value differences of foraging versus waiting; positive trial-by-trial relation)						
Occipital lobe	R	26	-96	5	519	7.15
Supramarginal gyrus	L	-63	-27	36	155	6.10
Mid-cingulate cortex	L/R	2	14	29	557	5.31
Perigenual ACC extending into VMPFC	L/R	6	50	6	221	4.53
**Optimal policy** (value differences of foraging versus waiting; negative trial-by-trial relation)						
Precuneus	R	29	-51	9	496	5.49
Supplementary motor area (SMA)	R	8	-29	65	277	4.97
**Choice uncertainty of heuristic: Uncertainty of probability of foraging success** (positive trial-by-trial relation with higher numbers indicating higher uncertainty)						
Occipital lobe/ superior parietal gyrus	R	30	-86	15	5196	8.91
Occipital lobe	L	-27	-89	15	1239	7.82
Superior parietal gyrus	L	-21	-63	56	1842	7.69
DLPFC	R	26	3	51	182	4.98
**Choice uncertainty of heuristic: Uncertainty of probability of foraging success** (negative trial-by-trial relation)						
Angular gyrus/ supramarginal gyrus/ middle temporal gyrus	R	60	-54	29	5852	8.36
Posterior cingulate cortex	L/R	15	-27	39	3719	7.63
IFG	R	48	35	-5	1697	7.37
Putamen extending into Rolandic operculum	R	26	2	6	1178	7.11
Occipital lobe (cuneus)	L/R	2	-83	32	4928	7.02
Perigenual ACC/ VMPFC	L/R	9	59	-2	5722	6.24
Insula	R	38	3	-11	348	6.03
Angular gyrus	L	-41	-77	36	1108	5.85
Middle temporal gyrus	L	-56	-63	2	331	5.71
Dorsal MPFC	L/R	3	56	33	252	4.50
**Choice uncertainty of optimal policy** (positive trial-by-trial relation with higher numbers indicating higher uncertainty)						
Putamen	R	29	6	12	158	5.79
Lingual gyrus	R	8	-50	0	140	4.86

Middle occipital gyrus	L	-41	-65	8	144	4.79
Superior temporal gyrus	L	-44	-11	-6	134	4.70
**Choice uncertainty of optimal policy** (negative trial-by-trial relation)						
IFG	R	48	26	-9	1234	5.88
DMPFC extending into ACC	R	6	42	38	184	4.81
**Discrepancy between heuristic and optimal policies** (positive trial-by-trial relation with higher numbers indicating larger discrepancies)						
DMPFC/ pre-SMA/ ACC	L/R	-9	36	38	14021	8.25
Dorsal striatum	R	11	20	-3	866	7.66
Dorsal striatum	L	-11	12	3	736	7.16
IFG	R	50	26	-11	1265	6.85
Middle temporal gyrus	R	59	-24	-8	335	6.67
IFG	L	-30	26	-5	552	6.17
Supramarginal sulcus	R	56	-47	29	1756	5.67
Midbrain	L/R	0	-30	-2	365	5.44
Supramarginal sulcus	L	-53	-54	45	371	5.10
Posterior cingulate cortex	L/R	5	-23	36	203	4.77
**Discrepancy between heuristic and optimal policies** (negative trial-by-trial relation)						
Rolandic operculum	R	59	2	6	295	5.91
Rolandic operculum	R	48	-30	21	671	5.55
Postcentral gyrus	R	17	-47	71	346	5.41
Posterior mid-cingulate cortex	R	12	-11	45	192	5.40
Superior temporal gyrus	L	-50	-35	21	289	5.21
Postcentral gyrus	L	-17	-44	69	139	4.94
Middle occipital gyrus	L	-41	-74	5	337	4.77

Clusters are whole-brain FWE corrected for multiple comparisons at $p < 0.05$ with a cluster-defining threshold of $p < 0.001$. See Fig. 6 and Fig. 7 for depiction of the most important clusters.

Supplementary Table 8. fMRI results during choice phase (GLM with participants' choices plus all relevant variables as parametric modulators)

	Side	Peak voxel MNI coordinates (mm)			Cluster size (Voxel)	Peak t score
		x	Y	z		
**Heuristic: Probability of foraging success** (positive trial-by-trial relation with higher numbers indicating higher probability)						
Dorsal MPFC (DMPFC) extending into pre-supplementary motor area (pre-SMA)	L/R	12	26	47	237	6.13
Intraparietal sulcus (IPS)	R	51	-56	45	2314	5.97
Dorsolateral cortex (DLPFC)	R	33	21	59	1126	5.62
Paracentral lobule	L/R	6	-30	60	802	5.26
Frontal pole (middle frontal gyrus)	L	-48	44	-6	413	4.78
**Heuristic: Probability of foraging success** (negative trial-by-trial relation)						
Perigenual anterior cingulate cortex (ACC)/ ventral MPFC (VMPFC)	L/R	-5	41	8	330	5.12
**Optimal policy** (value differences of foraging versus waiting; positive trial-by-trial relation)						
Mid-cingulate cortex	L/R	2	14	29	504	5.19
Occipital lobe	R	29	-93	3	202	4.88
Occipital lobe	R	15	-90	23	159	4.72
Perigenual ACC extending into VMPFC	L/R	5	38	17	183	4.68
**Optimal policy** (value differences of foraging versus waiting; negative trial-by-trial relation)						
None						
**Choice uncertainty of heuristic: Uncertainty of probability of foraging success** (positive trial-by-trial relation with higher numbers indicating higher uncertainty)						
Occipital lobe/ superior parietal gyrus	R	30	-90	11	4647	8.58
Occipital lobe	L	-27	-89	15	1152	7.29
Superior parietal gyrus	L	-21	-65	59	1367	6.58
**Choice uncertainty of heuristic: Uncertainty of probability of foraging success** (negative trial-by-trial relation)						
Angular gyrus/ supramarginal gyrus/ middle temporal gyrus	R	60	-54	29	4384	8.34
Posterior cingulate cortex	L/R	15	-27	39	2778	7.82
IFG	R	48	35	-5	1782	7.65
Occipital lobe (cuneus)	L/R	2	-80	27	4137	7.32
Putamen (extending into Rolandic operculum)	R	30	8	3	1112	6.62
Perigenual ACC/ VMPFC	L/R	9	59	0	5092	6.18
Insula	R	42	5	-14	312	5.85
Angular gyrus	L	-41	-77	36	1162	5.78
Middle temporal gyrus	L	-59	-63	3	360	5.72
Dorsal MPFC	L/R	-24	26	57	255	4.50
**Choice uncertainty of optimal policy** (positive trial-by-trial relation with higher numbers indicating higher uncertainty)						
None						
**Choice uncertainty of optimal policy** (negative trial-by-trial relation)						
IFG	R	48	26	-9	577	5.14
**Discrepancy between heuristic and optimal policies** (positive trial-by-trial relation with higher numbers indicating larger discrepancies)						
DMPFC/ pre-SMA/ ACC	L/R	-11	35	38	12862	7.91
Dorsal striatum	L	-11	14	5	591	7.39
Dorsal striatum	R	12	21	-3	636	6.82
Supramarginal sulcus	R	56	-47	29	2181	6.46
IFG	L	-30	26	-5	507	5.93

IFG	R	50	26	-11	997	5.79
Middle temporal gyrus	R	59	-24	-8	161	5.25
DLPFC	L	-33	27	42	217	5.16
Midbrain	L/R	2	-29	0	324	5.16
Posterior cingulate cortex	R	17	-45	36	218	5.03
Supramarginal sulcus	L	-53	-54	45	496	4.99
Posterior cingulate cortex	L/R	6	-24	36	308	4.89
**Discrepancy between heuristic and optimal policies** (negative trial-by-trial relation)						
Postcentral gyrus	R	17	-44	59	244	5.10
Middle occipital gyrus	L	-41	-75	5	298	5.04
Rolandic operculum	R	59	2	6	158	4.89
Rolandic operculum	R	50	-26	18	236	4.69
**Participants' choices** (binary parametric modulator: foraging > waiting)						
DLPFC	L	-26	-5	57	1103	7.87
Occipital lobe (middle occipital gyrus)	R	26	-96	6	593	7.27
DLPFC	R	21	2	56	656	7.20
Superior parietal gyrus	L	-18	-68	51	1887	6.99
Occipital lobe (middle occipital gyrus)	L	-29	-93	6	410	6.08
IFG	L	-30	20	9	522	5.94
Superior parietal gyrus	R	18	-57	63	999	5.42
IFG	R	32	20	-6	604	5.07
DLPFC	L	-50	5	30	263	4.76
**Participants' choices** (binary parametric modulator: waiting > foraging)						
Occipital lobe	L/R	2	-78	29	23214	11.06
Superior temporal gyrus	L	-62	-6	-5	870	6.06
Angular gyrus	L	-41	-74	39	913	5.53
Postcentral gyrus	L	-18	-41	60	1740	5.44
DLPFC	R	24	27	41	368	5.43
Orbitofrontal cortex	L	-26	45	0	239	5.41
DLPFC	L	-23	33	32	293	5.32
Rolandic operculum extending into superior temporal gyrus	R	50	-23	12	1937	5.18
Putamen	L	-29	-20	6	333	5.15

Clusters are whole-brain FWE corrected for multiple comparisons at $p < 0.05$ with a cluster-defining threshold of $p < 0.001$.

Supplementary Table 9. fMRI results during choice phase (GLM with participants' choices as only parametric modulator)

	Side	Peak voxel MNI coordinates (mm) x	Y	z	Cluster size (Voxel)	Peak t score
**Participants' choices** (binary parametric modulator: foraging > waiting)						
Occipital lobe (middle occipital gyrus)	R	30	-93	5	831	8.64
Dorsolateral cortex (DLPFC)	L	-24	-3	60	1543	8.30
Superior parietal gyrus	L	-20	-69	56	3946	8.05
Dorsal MPFC (DMPFC) extending into perigenual anterior cingulate cortex (ACC)	L/R	8	38	42	1515	7.47
Dorsal striatum	R	8	12	6	765	7.23
Inferior frontal gyrus (IFG)	R	29	29	0	1419	7.05
Midbrain extending into dorsal striatum	L/R	-5	-24	-5	2343	6.78
DLPFC	L	-45	6	27	1018	6.66
Superior parietal gyrus	R	17	-60	63	1728	6.23
IFG	L	-30	21	6	795	5.96
DLPFC	R	23	0	56	1030	5.59
**Participants' choices** (binary parametric modulator: waiting > foraging)						
Occipital lobe extending into posterior cingulate cortex (PCC)	L/R	-2	-81	26	38386	17.96
Middle occipital gyrus	R	48	-75	3	354	7.10
Rolandic operculum extending into superior temporal gyrus	R	66	-24	20	6256	7.08
Hippocampus	R	23	-18	-17	314	6.59
Rolandic operculum extending into superior temporal gyrus	L	-45	-17	0	3843	6.24
Orbitofrontal cortex	L	-21	47	-3	578	6.02
Angular gyrus	L	-57	-62	24	1728	5.47
DLPFC	L	-17	39	35	992	5.38
Orbitofrontal cortex	R	21	42	-2	425	5.29

Clusters are whole-brain FWE corrected for multiple comparisons at $p < 0.05$ with a cluster-defining threshold of $p < 0.001$. See **Supplementary Fig. 14** for depiction of the most important clusters.

Supplementary Table 10. fMRI results during choice phase (GLM with values of chosen options as parametric modulator)

	Side	Peak voxel MNI coordinates (mm)			Cluster size (Voxel)	Peak t score
		X	Y	z		
**Chosen option according to heuristic: Probability of foraging success** (positive trial-by-trial relation with higher numbers indicating higher probabilities of the chosen option)						
Posterior middle temporal gyrus	R	53	-63	9	952	6.50
Superior parietal gyrus extending into postcentral gyrus	R	15	-45	62	1242	5.86
Occipital lobe (cuneus)	L/R	12	-80	23	1175	5.80
Posterior middle temporal gyrus	L	-59	-62	2	287	5.52
Supramarginal gyrus	R	51	-33	35	1888	5.28
Superior parietal gyrus extending into postcentral gyrus	L	-18	-44	66	479	4.99
Frontal pole (inferior and middle frontal gyrus)	L	-47	39	6	212	4.63
**Chosen option according to optimal policy** (positive trial-by-trial relation with higher numbers indicating higher values of the chosen option)						
Putamen	R	20	6	-11	247	5.59
Supplementary motor area (SMA)	L/R	-6	-8	57	815	5.44
Ventral MPFC (VMPFC) extending into perigenual ACC	L/R	2	63	11	355	4.76

Clusters are whole-brain FWE corrected for multiple comparisons at $p < 0.05$ with a cluster-defining threshold of $p < 0.001$. See **Supplementary Fig 15** for depiction of the most important clusters.

Supplementary Table 11. fMRI results during outcome and forest phases (main GLM)

	Side	Peak voxel MNI coordinates (mm)			Cluster size (Voxel)	Peak t score
		x	Y	z		
**Outcome phase: Difference between current and past energy states** (positive trial-by-trial relation with higher numbers indication higher current than past states)						
Posterior cingulate cortex/ occipital lobe/ precuneus / striatum/ superior temporal sulcus/ VMPFC	L/R	0	-26	48	75402	9.01
MPFC/ DLPFC	L	-17	35	44	1553	5.86
Inferior frontal gyrus	R	30	35	-9	220	4.55
**Outcome phase: Difference between current and past energy states** (negative trial-by-trial relation)						
DMPFC	R	6	29	54	181	4.46
Anterior insula	R	41	23	-3	483	5.10
**Forest phase: Initial energy state** (positive trial-by-trial relation with higher numbers indicating higher energy states)						
Precuneus	R	15	-53	32	170	5.00
**Forest phase: Initial energy state** (negative trial-by-trial)						
None						
**Forest phase: Overall starvation probability** (positive trial-by-trial relation with higher numbers indicating higher starvation probability)						
None						
**Forest phase: Overall starvation probability** (negative trial-by-trial relation)						
Insula	L	-39	14	12	1522	7.03

Clusters are whole-brain FWE corrected for multiple comparisons at $p < 0.05$ with a cluster-defining threshold of $p < 0.001$. See **Supplementary Fig. 16** for depiction of the most important clusters.

Supplementary Note 1: Task instructions (translated into English)

Computer game: **Hunter and Gatherer**
Imagine that you are a hunter and gatherer. Imagine that you live in a world without food stores and supermarkets.

<u>Energy level:</u>

- You need food every day. In the game, an energy bar depicts your current energy level (similar to many computer games).

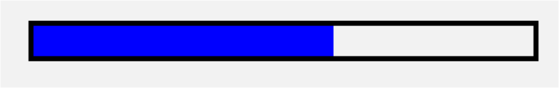

- If you have lost, all energy points you are "dead" in the game.

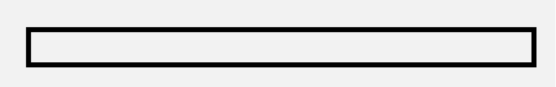

- If the bar is maximally filled, you have reached the maximal energy level in the game. That is, **five** energy points.

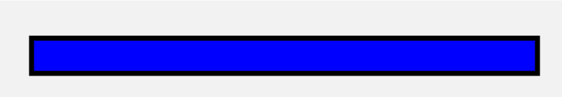

- Your final payment depends on whether you have died or not. More details will follow.

<u>Hunting & gathering:</u>

- To avoid starvation, you can go hunting or gathering food on every day. In the game, you have to decide whether you want to "hunt" ("Jagen") or to "wait" ("Warten").
- If you wait, you lose one energy point for sure. Depicted by a **red** dot. 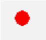
- Hunting has a risk: there is a probability that on some days hunting is NOT successful. In that case you lose **two** energy points. 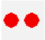 (For an unsuccessful hunt you lose one energy point more than for waiting because hunting consumes energy.)
- But: You can gain different numbers of energy points by hunting. With these points you can fill up your energy level. Different types of food provide different amounts of energy. These are depicted by **green** dots: e.g. 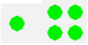

<u>Forest and weather:</u>

Imagine that you live in a certain “forest” and that you can hunt within that forest. Each forest has two different types of “weather:” good and bad weather. During the game, you are in different forests. In each forest, you start with a new amount of energy (i.e., “new live / new forest” = “Neues Leben / Neuer Wald”).
At the beginning of each forest, you see a screen that looks like the example below:

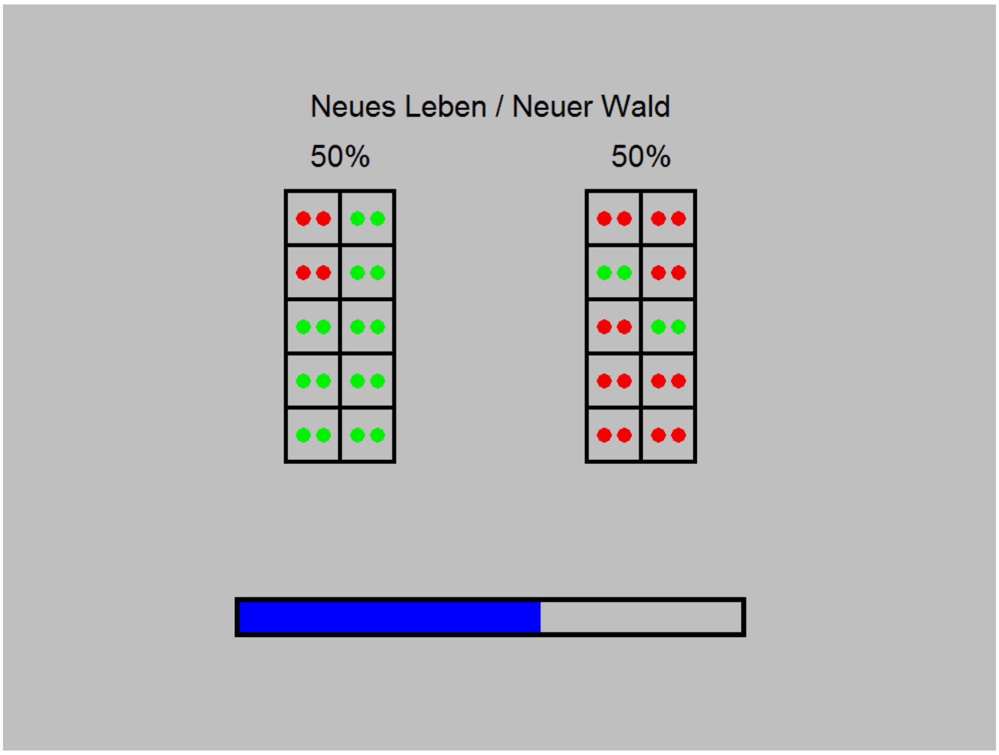

- The two “grids” depict how this forest looks like in good or bad weather.
- In the example above, the good weather is on the left and the bad weather is on the right.
- In the subfields of the two grids, the respective gains and losses of the forest are depicted.
- You will play several “days” in within this forest.
- On each day, good or bad weather occurs randomly with a probability of 50%.

Days:

As described above, you have to decide on each day, if you want to hunt ("Jagen") or to wait ("Warten").
This is one example screen:

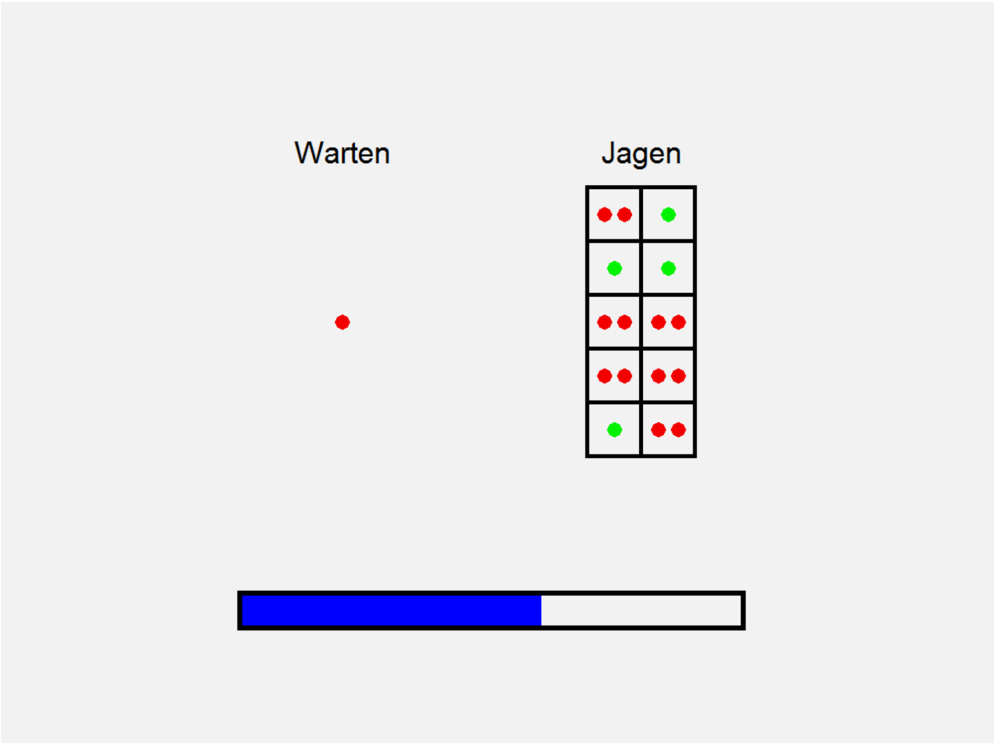

- If you hunt, one of the 10 subfields will be randomly selected and the corresponding gain will be added to your energy bar—or the corresponding loss will be deducted.
- For each decision, you have 2 sec. Please always try to answer in time. If you are too slow or if you press a wrong key, this will be indicated on the screen and **one** energy point will be deducted.

Here an example of how the outcome of the hunt will be depicted:
In this example, hunting was **not** successful. Two energy points were deducted, which led to starvation in this forest.

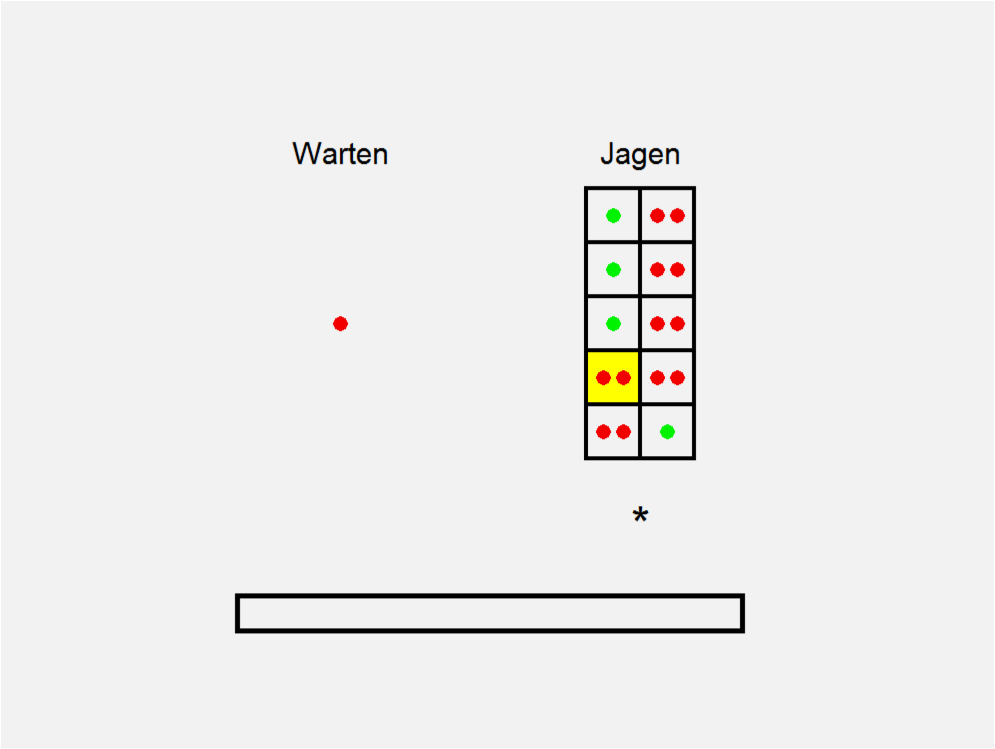

Here another example of how the outcome of the hunt will be depicted:
In this example, hunting was successful. Up to four energy points were added until the maximum amount of energy was reached.

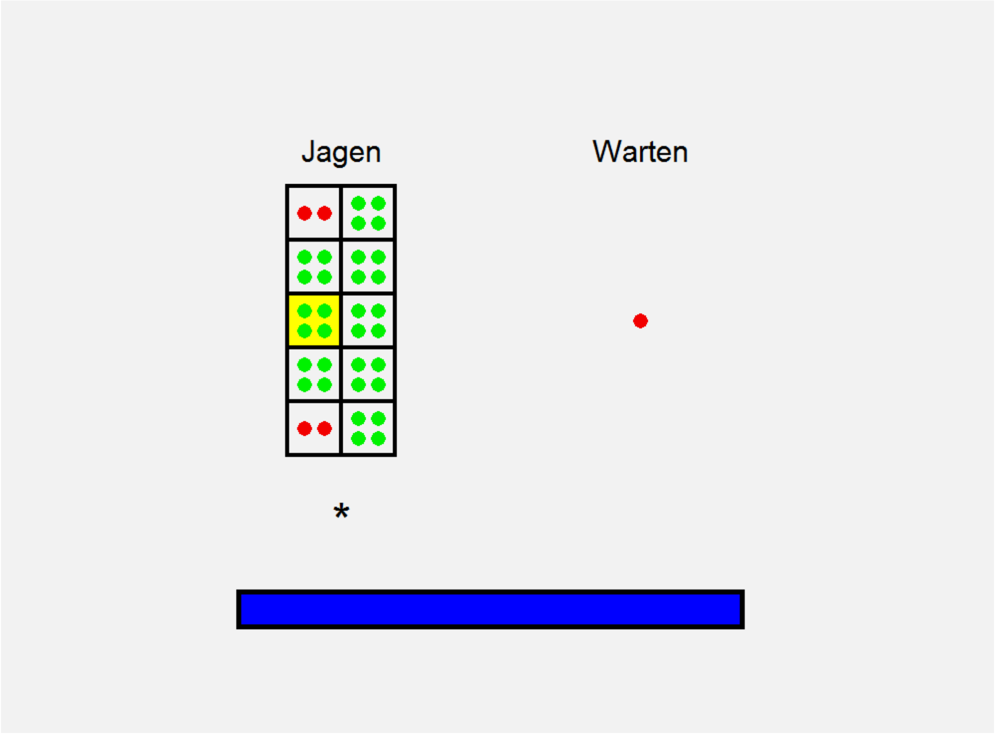

New Life / New Forest:

- Whenever you see the screen with the two types of weather, you get a “new life” in a new forest and your energy level is re-set.
- The different forests are independent of each other.
- Within each forest, you remain up to **five** days in a row. That is, your energy level on the following day depends on your decision at the current day. If you hunt, your energy level additionally depends on whether the hunt was successful or not. So, plan ahead in time.
- The days are not depicted directly.
- Since you remain **five** days in a row in a given forest, it is useful to pay attention to the depiction of the two weather types in the beginning.
- As may have noticed, you do not see a verbal indication whether the weather is good or bad on given day. You can only infer this from the possible gains and losses.
- If you have died in a forest, you have to press one of the two keys on the remaining number of days. Thus, the game is not shorter if you die.
- At the end of the experiment, you get money depending on whether you survived or not for **five** days within a forest.

Payment:

- You will play several blocks. We will randomly select one forest from each block.
- In the MR scanner, you sometimes play less than five days in one forest. If that was the case for the selected forest, you will play the remaining days outside the scanner to determine if you survived or not after **5 days**.
- The exact energy level is not important.
- For each of the selected forests in which you survived, you get CHF 1.50.

Key: You chose the left option with the left arrow key and the right option with the right arrow key.

Training: You will now do a short training session outside the scanner.

Questions: Please do not hesitate to ask questions.

Supplementary Note 2: Choice data according to prescriptions of different models
We specifically tested behavior in situations in which probability of foraging success and EV made opposing prescriptions. We chose these two metrics in particular since they are mathematically related and since previous studies have investigated how probability and EV account for choices in another type of sequential decision-making task [2,3]. We selected two types of trials: First, trials in which the heuristic of using the probability of foraging success prescribed foraging (i.e., model prescription > 0.5) and at the same time the heuristic of using EV prescribed waiting (i.e., model prescription < 0.5; based on logistic functions derived from mean parameter estimates from the behavioral sample). Second, trials in which the probability of foraging success prescribed waiting and the EV prescribed foraging. In both cases participants' actual choices were aligned with the prescriptions of the model including the probability of foraging success: In the first type of trials, participants were more likely to choose foraging (proportion of trials foraging chosen: mean ± SD: 0.73 ± 0.15, t-test against 0.5: $t(27) = 8.1$, $p < 10^{-7}$; percentage of these trials in the overall number of trials: 0.12 ± 0.01). In the second type of trials, participants chose waiting more often (proportion of trials foraging chosen: 0.11 ± 0.07, t-test against 0.5: $t(27) = -25.0$, $p < 10^{-19}$; percentage of these trials in the overall number of trials: 0.27 ± 0.02; see Supplementary Table 5 for analogous analyses comparing different horizons of the optimal policy).

Supplementary Note 3: IQ and questionnaires

For exploratory analyses, we also assessed participants verbal IQ using a vocabulary test implemented in the HAWIE-R, the German adaptation of the Wechsler Adult Intelligence Scale [1]. Participants' IQ scores were 101.6 ± 7.6 (mean ± SD in fMRI sample; n = 28) and 105.3 ± 9.9 (mean ± SD in behavioral sample; n = 21). No significant relationship between IQ scores and the parameter estimates for the optimal policy emerged in either sample (Pearson's correlations; p's > 0.1). To test this further, future work should use a more fine-grained assessment of intelligence and a more diverse sample.

Additionally, we administered a task specific questionnaire to assess how participants' rated the influence of the following task variables (mean ± SD across both samples; on a scale from 1 = no influence to 4 = strong influence): (a) probability of foraging success p: 3.4 ± 0.9; (b) magnitude of foraging gain g: 2.6 ± 0.8; (c) current energy state s: 3.5 ± 0.6; (d) current weather type: 3.1 ± 0.9; and (e) number of days past in a forest: 2.5 ± 1.0. In exploratory analyses, we correlated participants' ratings with the parameter estimates from the respective models. No significant relationships emerged (Bonferroni-corrected for conducting five Pearson's correlations; all p's > 0.1). We also asked participants (on a scale from 1 = never to 4 = always) whether they (f) actively counted the number of past days (2.3 ± 1.0) and whether they (g) were aware whether the current weather type was good or bad (3.0 ± 0.7).

Supplementary Note 4: Simulation of overall starvation probabilities

We performed simulations to derive stable probabilities of starvation in the used gambles (i.e., the 240 forests) under different decision variables. For each decision variable, we simulated 10000 runs and averaged the proportion of trials that resulted in starvation. Simulations were run for five days within each forest.

A simulation that strictly following the *a priori* optimal policy resulted in an average starvation probability of 0.101 for all days across all forests. Random choice resulted in a starvation probability of 0.209. From the participants in the fMRI sample, we derived parameter estimates of the relevant choice models, averaged over the resulting logistic functions, and used these in the simulations. The resulting percentages of starvation from these simulations indicated that the heuristic of using the probabilities of foraging success constituted the best tested heuristic (Supplementary Fig. 12).

Supplementary Note 5: Potential reasons for using the best heuristic available
We deem it unlikely that our task instructions or our depiction of the task variables primed participants to focus specifically on the probabilities of foraging success. The written task instructions first introduced internal energy states and the magnitudes of the possible gains before mentioning the probabilities of foraging success and did not explain these variables in more detail than the other task variables (see Supplementary Note 1 for an English translation of the written task instructions). If at all, the use of colored dots and spatial grids to depict the probabilities of foraging success (instead of for example pie charts) put slightly more emphasis on the magnitudes of foraging gains (see Fig. 1). Nevertheless, future studies could explicitly vary how well different heuristics approximate the optimal policy to test whether humans can flexibly use the locally best heuristic.

Supplementary References

1. Schmidt, K. H. & Metzler, P. *Wortschatztest (WST)*. (Beltz Test GmbH, 1992).
2. Venkatraman, V., Payne, J. W., Bettman, J. R., Luce, M. F. & Huettel, S. A. Separate neural mechanisms underlie choices and strategic preferences in risky decision making. *Neuron* **62,** 593–602 (2009).
3. Venkatraman, V., Payne, J. W. & Huettel, S. A. An overall probability of winning heuristic for complex risky decisions: choice and eye fixation evidence. *Organ. Behav. Hum. Decis. Process.* **125,** 73–87 (2014).
4. Baayen, R. H., Davidson, D. J. & Bates, D. M. Mixed-effects modeling with crossed random effects for subjects and items. *J. Mem. Lang.* **59,** 390–412 (2008).
